# Risk factors for hepatitis C infection among adult patients in Kedah state, Malaysia: A case–control study

**DOI:** 10.1371/journal.pone.0224459

**Published:** 2019-10-29

**Authors:** Mohd Azri Mohd Suan, Salmiah Md Said, Poh Ying Lim, Ahmad Zaid Fattah Azman, Muhammad Radzi Abu Hassan

**Affiliations:** 1 Department of Community Health, Faculty of Medicine and Health Sciences, Universiti Putra Malaysia, Serdang, Selangor, Malaysia; 2 Clinical Research Centre, Ministry of Health Malaysia, Hospital Sultanah Bahiyah, Alor Setar, Kedah, Malaysia; 3 Department of Medicine, Ministry of Health Malaysia, Hospital Sultanah Bahiyah, Alor Setar, Kedah, Malaysia; University of Cincinnati College of Medicine, UNITED STATES

## Abstract

Hepatitis C infection is a global public health problem. This study was designed to identify the risk factors associated with hepatitis C infection among adult patients in Kedah state, Malaysia. A matched, hospital-based, case–control study was conducted at a tertiary hospital. Cases were adult (aged ≥ 18 years) patients with positive serology test results for hepatitis C virus antibody and detectable hepatitis C virus RNA from January 2015 to December 2018, and controls were age-, sex- and ethnicity-matched patients who were not infected with hepatitis C virus. Self-administered questionnaires were used to collect data on demographic characteristics and previous exposure to selected risk factors among the study participants. Associations between hepatitis C and demographic and risk factors were assessed using univariable and multivariable logistic regression analyses. A total of 255 case–control patient pairs were enrolled. The multivariable analysis indicated that having a history of blood or blood product transfusion before 1992 (adjusted odds ratio [AOR] = 6.99, 95% confidence interval [CI]: 3.73–13.81), injection drug use (AOR = 6.60, 95% CI: 3.66–12.43), imprisonment (AOR = 4.58, 95% CI: 1.62–16.40), tattooing (AOR = 3.73, 95% CI: 1.37–12.00), having more than one sexual partner (AOR = 2.06, 95% CI: 1.16–3.69), piercing (AOR = 1.71, 95% CI: 1.04–2.80), and having only secondary education (AOR = 1.92, 95% CI: 1.06–3.57) were independently associated with hepatitis C. No associations were found between health care occupation, needle-prick injury, surgical procedures, haemodialysis, acupuncture, cupping, or contact sports and hepatitis C infection. These findings demonstrate that hepatitis C risk is multifactorial. Having a history of blood or blood product transfusion before 1992, injection drug use, imprisonment, tattooing, having more than one sexual partner, piercing, and having only secondary education were associated with increased odds of hepatitis C.

## Introduction

Hepatitis C virus (HCV) infection has increasingly become a public health concern in both developed and developing countries. The global prevalence of HCV infection was 1.0% in 2015, with the highest proportion of HCV-infected individuals found in the Eastern Mediterranean and European regions [1]. The World Health Organization (WHO) reported that in 2015, 71 million persons were living with this chronic viral infection worldwide [1]. Individuals with HCV infection are usually unaware that they are infected because the clinical symptoms of hepatitis C are non-specific and mild in most cases [2]. Delayed detection and treatment of hepatitis C lead this disease to progress to cirrhosis, hepatocellular carcinoma and liver failure [2]. In 2013, viral hepatitis, mainly comprising hepatitis C and B infection, caused more mortality (1.45 million deaths) than did HIV (1.34 million deaths), tuberculosis (1.29 million deaths) or malaria (0.85 million deaths) [3,4].

Compared with other areas of Asia, the Southeast Asian region has a relatively high burden of HCV, with an overall prevalence of 1.6%, ranging from 0.8% in Indonesia to 2.7% in Thailand [5]. This region has also been reported to have the third most deaths attributable to viral hepatitis on the continent, following East Asia and South Asia [3]. Malaysia, an upper middle-income country with an estimated population of 32 million, has been reported to have an HCV prevalence of 1.5%, which equates to nearly 330,000 infected adults [5]. From 2005 to 2017, more than 18,000 HCV-infected patients were newly diagnosed, and 460 deaths were reported across the country [6–8].

The large burden of this chronic viral infection necessitates greater access to testing and treatment. One of the key elements suggested by WHO in the 2017 *Global Hepatitis Report* is improving screening strategies to detect undiagnosed infected persons [1]. Identifying risk factors plays a central role in designing effective screening strategies. Risk factors commonly associated with HCV infection include unsafe blood transfusion and medical procedures, working in health care settings, injection drug use and imprisonment. However, affordable HCV testing is currently limited, globally. Of the estimated 71 million HCV-infected persons worldwide, only 14 million (20%) have been diagnosed [1]. Additionally, treatment has reached a small fraction of these people: Only 1.1 million (7%) of those diagnosed with HCV have begun treatment. Although access to HCV treatment is improving, it remains limited, especially in middle- and low-income countries [1]. For instance, although sofosbuvir and daclatasvir are available in 25 public hospitals in Malaysia, the high cost of these direct-acting antivirals has imposed a financial burden on the public health care system and limited access to HCV treatment in Malaysia [9]. As of 2017, more than 12,000 HCV-infected patients in Malaysia were awaiting access to direct-acting antivirals [10].

In Malaysia, several studies on risk behaviours associated with contracting HCV have been conducted. However, most of these studies have been limited to high-risk populations, particularly drug users [11,12], haemodialysis patients [13], fishermen [14] and blood donors [15]. Moreover, a notable concern is that patients with HCV who have no known risk factors have been reported to make up as much as 8% to 42% of the total HCV-infected population [12,16–18]. For the present study, we incorporated several factors that are potentially associated with HCV infection that have not previously been well studied in the Malaysia population. These factors were working as a health care professional, high-risk behaviours (intranasal drug use, imprisonment and men who have sex with men) and common traditional practices (acupuncture, cupping, home birth and male circumcision). We hypothesized that these sociodemographic characteristics, occupational profile, medical history, high-risk behaviours and traditional practices would be associated with an increased risk of HCV infection.

## Materials and methods

### Study site and ethics statement

This matched, hospital-based, case–control study was conducted at Hospital Sultanah Bahiyah (HSB) in Kedah, which is a government-funded tertiary hospital and one of 25 referral centres for HCV infection in Malaysia. Kedah, a state in northern Malaysia with a population of two million, was chosen as a study site because this state has a high number of HCV infection cases [7]. From 2013 to 2015, the incidence of HCV infection in Kedah ranged from 7.1 to 12.9 per 100,000 population. The Medical Research and Ethics Committee of the Ministry of Health of Malaysia approved this study (NMRR-17-3322-38795).

### Patient enrolment and matching criteria

Patients in both the case and the control group were enrolled from June to December 2018. These patients were adult (≥ 18 years of age) Malaysian citizens seeking treatment at HSB. Cases were those who had positive serology test results for HCV antibody and detectable HCV RNA from January 2015 to December 2018 and were followed up at the gastroenterology specialist outpatient clinic. These patients were identified using the Hepatitis Register Book, which was created at HSB in 2009 and is kept at the gastroenterology clinic. Information on year of diagnosis and serology results were cross-checked with the hospital electronic medical record system. Cases were approached after they had seen the attending physician for a scheduled follow-up visit. The purpose and method of the study were explained to the eligible cases by the first author, who is a medical doctor, before obtaining their informed consent. Each eligible case was matched with a control (1:1) by age (± 5 years), sex and ethnicity.

Controls were recruited among patients who attended other specialist outpatient clinics in the same hospital building. Because the gynaecology, obstetrics and psychiatric clinics were located in a different building, patients from these clinics were not selected. Potential control patients were identified from a computer-generated clinic appointment list for the same day as the case was identified. If the identified control patient did not fulfil the study criteria, the next matched control patient in the list was selected. Selection consisted of three steps: (i) identification of eligible control patients from the appointment list using the matching criteria; (ii) the first author approaching the patient in the waiting area outside the clinic and explaining the study; and (iii) obtaining informed consent and performing a blood screening for HCV antibody to confirm uninfected status. These blood samples were sent to a medical lab for anti-HCV antibody detection using a commercial fourth-generation enzyme immunoassay Nanbase C-96 V4.0 anti-HCV test (General Biological Corporation, Taiwan, China). Control patients with positive results were excluded.

A self-administered questionnaire was administered to the participants (both cases and controls) after they had provided informed consent. To minimise data incompleteness because of the sensitive questions asked in the questionnaire (regarding illicit drug use, HIV co-infection and risky sexual behaviours), the survey was completed in a private room at the clinic, and only one patient was allowed to be in the room at a time. Participants were asked to answer all questions but allowed to skip if they do not willing to disclose any personal information. Participants also informed that their information from the questionnaire would be handled in a confidential manner.

### Study instrument and variables

A paper-based, self-administered questionnaire was developed based on the literature on the risk factors for HCV infection. The questionnaire was written in both English and *Bahasa Malaysia* (the national language of Malaysia) by three bilingual authors. The dependent variable was hepatitis C infection status among the recruited patients. The risk factors assessed included the participants’ demographic characteristics (e.g. age, sex, ethnicity, education level and marital status), occupational profile (e.g. health care occupation, fishery and maritime work, unskilled work and unemployment), medical history (e.g. past surgical procedures, blood or blood product transfusion before 1992, needle-stick injury, HIV co-infection, born to an HCV-infected mother and history of evacuation of retained products of conception), involvement in high-risk behaviours (e.g. history of tattooing, injection drug use, intranasal drug use, history of imprisonment, history of undergoing cosmetic procedures, involvement in contact sports, having more than one sexual partner and men who have sex with men) and involvement in traditional practices (e.g. acupuncture, cupping therapy, piercing, male circumcision by a traditional practitioner and home birth with a traditional midwife). In total, there were 25 questions with ‘yes’ or ‘no’ response options regarding the patient’s exposure to specific risk factors. Information on patient’s age, sex and ethnicity was obtained from the patients’ national identity cards.

Recipients of blood or blood product transfusions before 1992 were at risk of HCV infection because pre-screening services for blood donors in Malaysia became available in mid-1992 [19]. Cosmetic procedures were defined as any procedures to improve personal appearance, including but not limited to epilation, plucking, peeling, pedicure and manicure [20]. Any sport activities in which the participants would necessarily come into bodily contact with others, such as rugby and boxing, were considered contact sports. Piercing was defined as puncturing or cutting any part of one’s body (e.g. nose, tongue, ear or genitals) to create an opening to insert rings, studs or other pieces of jewellery. Five sex-specific questions were answered only by male (men who have sex with men and male circumcision) or female (evacuation of retained products of conception, working as a female commercial sex worker and home birth with a traditional midwife) participants. The test–retest reliability of the questionnaire showed that the Cohen’s kappa coefficients for most of the questions reflected either excellent (kappa > 0.75) or good (kappa values of 0.40–0.75) agreement [21].

### Statistical analysis

The matching characteristics of the case and control groups are presented as frequencies and percentages. Differences in the matching characteristics were assessed using Pearson’s chi-squared test. Crude odds ratios and 95% confidence intervals (CIs) were determined for each potential risk factor using univariable logistic regression analysis, and the significance level was fixed at *p* < 0.05. The associations between HCV status and demographic characteristics, occupational profile, medical history, traditional practices and high-risk behaviours were subsequently assessed using multivariable logistic regression analysis. Unconditional logistic regression was used in this study because the data were matched on basic variables (age, sex and ethnicity) [22].

Except for the sex-related variables, all variables with a *p*-value < 0.25 in the univariable analysis were included in the multivariable model [23]. The variables in the final model were selected using stepwise procedures. The model was then checked for interactions between the included variables and for multicollinearity. Model fit was examined using the Hosmer–Lemeshow goodness-of-fit test, the classification table and the receiver operating characteristic curve. All analyses were performed using R statistical software, Version 3.5.2 [24]. The study methods and findings have been reported according to the recommendations of the Strengthening the Reporting of Observational Studies in Epidemiology (STROBE) guidelines.

## Results

During the data collection period, 16 case patients who were diagnosed before 2015 and four non-Malaysian patients (three in the case group and one in the control group) were excluded. Two patients who were not known to be HCV-infected but tested positive for anti-HCV antibody during the control group selection were also excluded. After excluding these patients, data from 255 matched case–control pairs remained for analysis. None of the patients had missing data. In both groups, most of the patients were men, aged 40 years or older and of Malay/Bumiputra ethnicity. There were no significant differences between the case and control groups in term of age group, sex or ethnicity (Table 1).

**Table 1:** Matching characteristics of the cases and controls.

Matching characteristic	Cases, n (%)		Controls, n (%)		*x*^2^ stat. (*df*) ^a^	*p*-value
Age groups					3.50 (4)	0.477
≤ 29	15	(5.9)	17	(6.7)		
30–39	36	(14.1)	45	(17.6)		
40–49	73	(28.6)	56	(22.0)		
50–59	75	(29.4)	79	(31.0)		
≥ 60	56	(22.0)	58	(22.7)		
Sex					0 (1)	1.000
Female	69	(27.1)	69	(27.1)		
Male	186	(72.9)	186	(72.9)		
Ethnicity					0 (2)	1.000
Indian	8	(3.1)	8	(3.1)		
Chinese	47	(18.5)	47	(18.5)		
Malay/Bumiputra	200	(78.4)	200	(78.4)		

^a^ Pearson’s chi-squared test; *df*: degrees of freedom

The frequencies of demographic characteristics, occupational profile, medical history, traditional practices and high-risk behaviours, as well as the univariable logistic regression results, are presented in Table 2. The results of the univariate analyses showed that having secondary, primary or no formal education; being an unskilled worker; history of blood transfusion before 1992; needle-prick injury; piercing; injection drug use; tattooing; history of imprisonment; and having more than one sexual partner were significantly associated with an increased risk of HCV infection. Twelve variables with *p*-values less than 0.25 were selected for the multivariable analyses. Two variables (men who have sex with men and home birth with a traditional midwife) were not selected because these variables were answered by only male or female participants.

**Table 2:** Univariable logistic regression analysis of risk factors for hepatitis C among adult patients in Kedah state.

Risk Factor	Cases, n (%)		Controls, n (%)		Crude OR (95% CI)		*p*-value
**Demographic characteristics**							
Educational level							
Higher education	22	(8.6)	53	(20.8)	1		
Secondary education	197	(77.3)	164	(64.3)	2.89	(1.71–5.04)	< 0.001*
Primary/no formal education	36	(14.1)	38	(14.9)	2.28	(1.17–4.53)	0.016*
Marital status							
Married/ever married	204	(80.0)	214	(83.9)	1		
Single	51	(20.0)	41	(16.1)	1.30	(0.83–2.06)	0.251
**Occupation**							
Health care worker							
No	250	(98.0)	243	(95.3)	1		
Yes	5	(2.0)	12	(4.7)	0.41	(0.13–1.11)	0.094
Fishery and maritime worker							
No	242	(94.9)	246	(96.5)	1		
Yes	13	(5.1)	9	(3.5)	1.47	(0.62–3.62)	0.386
Unskilled worker							
No	223	(87.5)	241	(94.5)	1		
Yes	32	(12.5)	14	(5.5)	2.47	(1.31–4.89)	0.007*
Unemployed							
No	173	(67.8)	170	(66.7)	1		
Yes	82	(32.2)	85	(33.3)	0.95	(0.65–1.37)	0.777
**Medical history**							
Blood/blood product transfusion before 1992							
No	204	(80.0)	240	(94.1)	1		
Yes	51	(20.0)	15	(5.9)	4.00	(2.24–7.56)	< 0.001*
Needle-prick injury							
No	237	(92.9)	248	(97.3)	1		
Yes	18	(7.1)	7	(2.7)	2.69	(1.15–7.03)	0.029*
Surgical procedure							
No	227	(89.0)	230	(90.2)	1		
Yes	28	(11.0)	25	(9.8)	1.13	(0.64–2.02)	0.663
Haemodialysis							
No	245	(96.1)	245	(96.1)	1		
Yes	10	(3.9)	10	(3.9)	1.00	(0.40–2.48)	1.000
HIV co-infection							
No	242	(94.9)	237	(92.9)	1		
Yes	13	(5.1)	18	(7.1)	0.71	(0.33–1.47)	0.356
Born to mothers with HCV infection							
No	252	(98.8)	254	(99.6)	1		
Yes	3	(1.2)	1	(0.4)	3.02	(0.38–61.35)	0.339
Evacuation of retained products of conception ǂ							
No	51	(73.9)	50	(72.5)	1		
Yes	18	(26.1)	19	(27.5)	0.93	(0.43–1.98)	0.848
**Traditional practices**							
Acupuncture							
No	243	(95.3)	236	(92.5)	1		
Yes	12	(4.7)	19	(7.5)	0.61	(0.28–1.28)	0.198
Piercing							
No	183	(71.8)	205	(80.4)	1		
Yes	72	(28.2)	50	(19.6)	1.61	(1.07–2.45)	0.023*
Cupping (bloodletting) therapy							
No	227	(89.0)	227	(89.0)	1		
Yes	28	(11.0)	28	(11.0)	1.00	(0.57–1.75)	1.000
Male circumcision by a traditional practitioner ±							
No	75	(40.3)	77	(41.4)	1		
Yes	111	(59.7)	109	(58.6)	1.05	(0.69–1.58)	0.833
Home delivery by a traditional midwife ǂ							
No	52	(75.4)	59	(85.5)	1		
Yes	17	(24.6)	10	(14.5)	1.93	(0.82–4.72)	0.137
**High-risk behaviours**							
Injection drug use							
No	167	(65.5)	238	(93.3)	1		
Yes	88	(34.5)	17	(6.7)	7.38	(4.33–13.25)	< 0.001*
Intranasal drug use							
No	239	(93.7)	240	(94.1)	1		
Yes	16	(6.3)	15	(5.9)	1.07	(0.52–2.24)	0.853
Tattooing							
No	224	(87.8)	250	(98.0)	1		
Yes	31	(12.2)	5	(2.0)	6.92	(2.88–20.54)	< 0.001*
History of imprisonment							
No	212	(83.1)	251	(98.4)	1		
Yes	43	(16.9)	4	(1.6)	12.73	(5.05–42.81)	< 0.001*
Cosmetic procedures							
No	247	(96.9)	246	(96.5)	1		
Yes	8	(3.1)	9	(3.5)	0.89	(0.33–2.35)	0.805
Spouse with HCV infection							
No	251	(98.4)	251	(98.4)	1		
Yes	4	(1.6)	4	(1.6)	1.00	(0.23–4.27)	1.000
Contact sports							
No	244	(95.7)	242	(94.9)	1		
Yes	11	(4.3)	13	(5.1)	0.84	(0.36–1.91)	0.676
More than one sexual partner							
No	198	(77.6)	229	(89.8)	1		
Yes	57	(22.4)	26	(10.2)	2.54	(1.55–4.24)	< 0.001*
Men who have sex with men ±							
No	185	(99.5)	180	(96.8)	1		
Yes	1	(0.5)	6	(3.2)	0.16	(0.01–0.96)	0.094
Women working as commercial sex worker ǂ							
No	66	(95.7)	69	100.0	-		
Yes	3	(4.3)	0	0.0	-		-

OR: odds ratio; CI: confidence interval; HCV: hepatitis C virus

* *p* < 0.05

ǂ Only female patients (n = 69 per group)

± Only male patients (n = 186 per group)

In the multivariable analysis, seven factors were significantly associated with HCV infection. Compared with adult patients with tertiary education, the odds of contracting HCV infection were higher among patients with only secondary education, with an adjusted odds ratio (AOR) of 1.92 (95% confidence interval [CI]: 1.06–3.57). Adult patients with a history of blood or blood product transfusion before 1992 had higher odds of having hepatitis C, compared with those without this history (AOR = 6.99, 95% CI: 3.73–13.81). Compared with those without piercings, adult patients with piercings were more likely to have HCV infection (AOR = 1.71, 95% CI: 1.04–2.80). Similarly, patients with tattoos had higher odds of having HCV, compared with those without tattoos (AOR = 3.73, 95% CI: 1.37–12.00). History of imprisonment was another risk factor for hepatitis C (AOR = 4.58, 95% CI: 1.62–16.40). The odds of having HCV infection were higher among patients who used injection drugs (AOR = 6.60, 95% CI: 3.66–12.43) than among those who did not use injection drugs. Adult patients with more than one sexual partner were at higher odds of having hepatitis C than were patients with one sexual partner (AOR = 2.06, 95% CI: 1.16–3.69) (Table 3).

**Table 3:** Factors independently associated with hepatitis C infection among adult patients in Kedah state.

Variable	Adj. OR ^±^	(95% CI)	*p*-value ^ǂ^
Educational level			
Higher Education	1		
Secondary education	1.92	(1.06, 3.57)	0.034*
Primary/no formal education	1.23	(0.57, 2.70)	0.595
Blood or blood product transfusion before 1992			
No	1		
Yes	6.99	(3.73, 13.81)	<0.001*
Piercing			
No	1		
Yes	1.71	(1.04, 2.80)	0.033*
Tattooing			
No	1		
Yes	3.73	(1.37, 12.00)	0.016*
History of imprisonment			
No	1		
Yes	4.58	(1.62, 16.40)	0.008*
Injection drug use			
No	1		
Yes	6.60	(3.66, 12.43)	<0.001*
Having more than one sexual partner			
No	1		
Yes	2.06	(1.16, 3.69)	0.013*

Adj. OR: adjusted odds ratio; CI: confidence interval

± Multivariable logistic regression (The model fit was reasonably good, the model assumptions were met, there were no multicollinearity problems and there were no significant interactions between the variables.)

ǂ Likelihood-ratio test

* *p* < 0.05

## Discussion

This is one of the first studies using the matched case–control design to investigate the risk factors for HCV infection in Malaysia. In this study, the risk factors identified included having a history of blood or blood product transfusion before 1992, injection drug use, imprisonment, tattooing, piercing, having more than one sexual partner and having only secondary education. Contrary to our expectations, health care occupation, needle-prick injury, surgical procedures, haemodialysis, acupuncture, and cupping were not associated with HCV risk.

Health care facilities were previously considered a significant source of HCV, mainly because of a lack of injection safety, unsafe blood transfusion and risky medical procedures [1]. In Egypt, unsafe therapeutic injection and medical stitches were the two main health care-related factors associated with HCV infection [25]. The association of HCV infection with invasive procedures in hospital setting has also been described [26]. In the current study population, having a blood or blood product transfusion before 1992 posed the highest risk. This is due to the pre-screening services for blood donors in Malaysia which made available in mid-1992 and recipients of blood or blood product transfusions before 1992 were at risk of HCV infection [19]. In contrast to previous findings [20, 26–28], other medical exposures such as surgical procedures, haemodialysis, HIV co-infection and vertical transmission were not found to be significantly associated with HCV infection. Although needle-prick injury showed significant association in univariable analysis, this association was not established in multivariable analysis. Safer medical procedures may be attributed to the continuous efforts made by the Ministry of Health of Malaysia to control the nosocomial transmission of HCV in health care facilities. Because blood transfusion and medical procedures are becoming safer, the main risk of HCV infection is now posed by injection drug use.

Drug abuse has been a major concern in Malaysia for the past few decades. In 2017, more than 7000 drug users were recorded in northern Malaysia, which includes Kedah state [29]. Opiates are the most widely abused type of drug in the country, and needles are often shared among drug users, which is the main mode of transmission in this group [29,30]. Injection drug use has consistently appeared as a leading factor in HCV infection and transmission in several studies conducted in Malaysia [11,12,16,17]. A previous study also found that the prevalence of HCV infection was higher among injection drug users (67.1%) than among non-injection drug users (30.8%) [11]. Among upper middle-income countries, the prevalence of HCV among injection drug users in Malaysia is comparable to prevalence in Montenegro and Brazil, although lower than China [31]. This large population of drug users, combined with the high prevalence of HCV among this group, warrants greater efforts to address this issue. Better access to sterile injection equipment and community-led health campaigns to promote public awareness of the unsafe use of illicit drugs are proposed to strengthen the existing methadone replacement therapy and needle-exchange programmes in Malaysia. User involvement in peer-based programmes has also been shown to be effective in increasing HCV awareness among injection drug users [32].

Patients with a history of imprisonment were another group found to have higher odds of HCV infection. Prisons are generally high-risk environments for the transmission of hepatitis and other infectious diseases [33]. This situation is mainly attributed to factors inside prisons (e.g. overcrowding and poor sanitation) and the risky lifestyles of prisoners, either before or while they are in prison. Moreover, the prevalence rates of injection drug use, tattooing, piercing and risky sexual behaviours have been reported to be high among prisoners worldwide [34], which may increase the chance of contracting this viral infection. However, data on HCV infection among prisoners in Malaysia are limited because HCV screening policies do not exist upon prison entry and thereafter. With more than 55,000 inmates reported nationwide in Malaysia [35], prisons may be an important site for the spread of HCV in the country. Future studies should focus on identifying specific source of HCV infection among Malaysian prisoners.

This study demonstrates the role of piercing and tattooing in HCV transmission in this population. Participants with tattoo or piercing were at risk of HCV due to repeated use of tattoo or piercing instruments that undergone improper sterilisation. Moreover, the percentage of HCV infected patients with tattoo (12.2%) and piercing (28.2%) in this study is considerably high in comparison with previous local studies [12,16,18], which indicates that both practices have become increasingly popular among studied population. Hence, formulating safe practice guidelines for piercing and tattooing are urgently needed to curb the HCV infection.

Higher odds of HCV infection were found among patients with more than one sexual partner although infection through sexual exposures is less common. However, this finding is not consistent with other studies [36]. While this study also identifies educational level as a risk factor for HCV infection, reason that may have contributed to higher HCV risk among participants with secondary education is unclear and require further studies to support this association.

In previous studies, maritime and health care workers have been identified as having relatively high HCV risk [37, 38]. However, the present study did not find an association between these occupations and HCV infection. It is noteworthy that the small numbers of patients in these two occupation groups in the present study could have resulted in these inconsistent findings. Likewise, traditional practices such as acupuncture, cupping, home birth and male circumcision were not associated with increased odds of HCV infection in the present study.

This study had several potential limitations. First, the data on the exposure were collected retrospectively, and recall bias is inevitable. Therefore, the accuracy of the data was subject to the patients’ ability to recall past exposure. Second, the study participants were recruited from hospital which may introduce selection bias as participants from hospital may have different characteristics from general population. For instance, controls selected from hospital patients may relatively have higher possibility of kidney disease requiring haemodialysis and HIV co-infection than the population. Therefore, the estimates of the risk exposure among controls may differ from that in the population and subsequently result in a biased estimate of the association between risk factor and HCV. Third, despite our efforts to conduct the survey in a private room with only one patient at a time present, some patients may not have disclosed their involvement in high-risk activities or their full medical history, including injection drug use, HIV status and high-risk sexual relationships, because of concerns about stigma. This may have led to an underestimation of the participants’ actual exposure to such activities. Forth, the study participants were enrolled from a single centre, and the findings may not be generalisable to the general population. However, because the study site was a referral centre for hepatitis C, the recruited patients came from various hospitals across northern Malaysia, which partly improves the generalisability of the study results.

To conclude, this study demonstrates that the risk of HCV infection is multifactorial. In line with the WHO’s recommendation to enhance strategies to find undiagnosed infected persons, national screening activities should give greater attention especially to individuals with a history of blood or blood product transfusion before 1992, injection drug users, those who have been imprisoned, those with a history of tattooing, those involved in piercing, those with more than one sexual partner and those with only secondary education. Considering the chronic consequences of HCV infection, future risk reduction programmes should emphasise injection drug users and the prisoner population. More data also required to confirm the association between sexual behaviours, educational level and HCV infection.

## Supporting information

S1 Appendix STROBE checklist. (DOC)

S1 Dataset Dataset for study on risk factors for hepatitis C infection in Kedah (n = 510). (XLSX)
