# Supplementary material for: Risk factors for hepatitis C infection among adult patients in Kedah state, Malaysia: A case–control study
Source: PLoS One. 2019 Oct 29;14(10):e0224459. doi: 10.1371/journal.pone.0224459 (PMC6818779; doi:10.1371/journal.pone.0224459)
Supplement: S1 Appendix — (DOC) [file pone.0224459.s001.doc]

**S1 Appendix**

STROBE checklist of items included in reports of case-control studies.

|  | Item No | Recommendation | Page No. (in manuscript) | Remarks |
| --- | --- | --- | --- | --- |
| **Title and abstract** | 1 | (*a*) Indicate the study’s design with a commonly used term in the title or the abstract | 1 |  |
|  |  | (*b*) Provide in the abstract an informative and balanced summary of what was done and what was found | 2 |  |
| Introduction | | |  |  |
| Background/rationale | 2 | Explain the scientific background and rationale for the investigation being reported | 3-4 |  |
| Objectives | 3 | State specific objectives, including any prespecified hypotheses | 4 |  |
| Methods | | |  |  |
| Study design | 4 | Present key elements of study design early in the paper | 5 |  |
| Setting | 5 | Describe the setting, locations, and relevant dates, including periods of recruitment, exposure, follow-up, and data collection | 5-8 |  |
| Participants | 6 | (*a*) Give the eligibility criteria, and the sources and methods of case ascertainment and control selection. Give the rationale for the choice of cases and controls | 5-6 |  |
|  |  | (*b*) For matched studies, give matching criteria and the number of controls per case | 5 |  |
| Variables | 7 | Clearly define all outcomes, exposures, predictors, potential confounders, and effect modifiers. Give diagnostic criteria, if applicable | 6-8 |  |
| Data sources/ measurement | 8* | For each variable of interest, give sources of data and details of methods of assessment (measurement). Describe comparability of assessment methods if there is more than one group | 6-8 |  |
| Bias | 9 | Describe any efforts to address potential sources of bias | 18-19 |  |
| Study size | 10 | Explain how the study size was arrived at | 8 |  |
| Quantitative variables | 11 | Explain how quantitative variables were handled in the analyses. If applicable, describe which groupings were chosen and why | 8 |  |
| Statistical methods | 12 | (*a*) Describe all statistical methods, including those used to control for confounding | 8 |  |
|  |  | (*b*) Describe any methods used to examine subgroups and interactions | 8 | No subgroup analysis |
|  |  | (*c*) Explain how missing data were addressed | NA | No missing data, stated in ‘Result’ section. |
|  |  | (*d*) If applicable, explain how matching of cases and controls was addressed | 5, 8 |  |
|  |  | (*e*) Describe any sensitivity analyses | 8 |  |
| Results | | |  |  |
| Participants | 13* | (a) Report numbers of individuals at each stage of study—eg numbers potentially eligible, examined for eligibility, confirmed eligible, included in the study, completing follow-up, and analysed | 9 |  |
|  |  | (b) Give reasons for non-participation at each stage | 9 |  |
|  |  | (c) Consider use of a flow diagram | NA |  |
| Descriptive data | 14* | (a) Give characteristics of study participants (eg demographic, clinical, social) and information on exposures and potential confounders | 9 |  |
|  |  | (b) Indicate number of participants with missing data for each variable of interest | NA | Of all recruited patients, no missing data was found. Stated in ‘Result’ section. |
| Outcome data | 15* | Report numbers in each exposure category, or summary measures of exposure | 9-10 | Table 1 |
| Main results | 16 | (*a*) Give unadjusted estimates and, if applicable, confounder-adjusted estimates and their precision (eg, 95% confidence interval). Make clear which confounders were adjusted for and why they were included | 10-12 | Table 2, Table 3 |
|  |  | (*b*) Report category boundaries when continuous variables were categorized | 9 | Only variable age was categorized. |
|  |  | (*c*) If relevant, consider translating estimates of relative risk into absolute risk for a meaningful time period | NA | Not relevant to this study |
| Other analyses | 17 | Report other analyses done—eg analyses of subgroups and interactions, and sensitivity analyses | 13-14 | Interaction between variables, sensitivity analyses, Hosmer-Lemeshow goodness-of-fit test, classification table, and receiver operating characteristic (ROC) curve. |
| **Discussion** | | |  |  |
| Key results | 18 | Summarise key results with reference to study objectives | 15 |  |
| Limitations | 19 | Discuss limitations of the study, taking into account sources of potential bias or imprecision. Discuss both direction and magnitude of any potential bias | 18-19 |  |
| Interpretation | 20 | Give a cautious overall interpretation of results considering objectives, limitations, multiplicity of analyses, results from similar studies, and other relevant evidence | 15-19 |  |
| Generalisability | 21 | Discuss the generalisability (external validity) of the study results | 18-19 |  |
| **Other information** | | |  |  |
| Funding | 22 | Give the source of funding and the role of the funders for the present study and, if applicable, for the original study on which the present article is based | NA | Stated in a separate ‘Financial Disclosure Statement’ |

*Give information separately for cases and controls.
